# Transarterial chemoembolization combined with apatinib in the treatment of advanced hepatocellular carcinoma: a prospective, multi-center, real-world study (Ahend-HAP02)

**DOI:** 10.3389/fonc.2025.1615911

**Published:** 2025-07-03

**Authors:** Hang Yuan, Zhen Li, Guang-Shao Cao, Fei Xu, Gang Wu, Peng-Xu Ding, Qiu-Liang Wei, Zu-Kuan Chang, Cheng Xing, Huan-Zhang Niu, Jun Yin, Quan-Jun Yao, Lin Zheng, Jin-Cheng Xiao, Cheng-Shi Chen, Hong-Tao Cheng, Deng-Wei Zong, Wei-Li Xia, Xiang Geng, Xiao-Hui Zhao, Hai-Liang Li, Hong-Tao Hu

**Affiliations:** ^1^ Department of Interventional Radiology, The Affiliated Cancer Hospital of Zhengzhou University & Henan Cancer Hospital, Zhengzhou, China; ^2^ Department of Interventional Radiology, The First Affiliated Hospital of Zhengzhou University, Zhengzhou, China; ^3^ Department of Intervention, Henan Provincial People’s Hospital, People’s Hospital of Zhengzhou University, Zhengzhou, China; ^4^ Department of Interventional Therapy, National Cancer Center/National Clinical Research Center for Cancer/Cancer Hospital, Chinese Academy of Medical Sciences and Peking Union Medical College, Beijing, China; ^5^ Department of Minimal-Invasive Intervention, Anyang Tumor Hospital/The Forth Affiliated Hospital of Henan University of Science and Technology, Anyang, China; ^6^ Department of Interventional Therapy, Xinxiang Central Hospital/the Fourth Clinical College of Xinxiang Medical University, Xinxiang, China; ^7^ Department of Interventional Therapy, Zhoukou Central Hospital, Zhoukou, China; ^8^ Department of lnterventional Radiology, The First Afiliated Hospital of Henan Science and Techology University, Luoyang, China; ^9^ Department of lnterventional Radiology, Xinyang Central Hospital, Xinyang, China; ^10^ Department of Interventional Therapy, Tianjin Medical University Cancer Institute & Hospital, National Clinical Research Center for Cancer, Tianjin, China

**Keywords:** transarterial chemoembolization, apatinib, hepatocellular carcinoma, real world, overall survival

## Abstract

**Purpose:**

To analyze the efficacy and safety of transarterial chemoembolization (TACE) with apatinib (TACE-A) for the treatment of advanced hepatocellular carcinoma (HCC).

**Methods:**

Data from advanced HCC patients treated with TACE-A between January 2019 and June 2022 were evaluated. The patients from 8 medical centers were included. The primary endpoints were overall survival (OS) and progression-free survival (PFS). The secondary endpoints were objective response rate (ORR) and adverse events (AEs). Prognostic factors affecting OS were analyzed, and the tumor imaging response at the first follow-up was evaluated to study the survival differences among patients.

**Results:**

A total of 389 patients were included, the median PFS was 7.0 months (95% confidence interval [CI]: 6.3–7.7), and the median OS (mOS) was 18.9 months (95% CI: 17.5–20.3). The median time of the first follow-up was 1.2 months, the ORR was 33.7%, and the mOS of the complete response, partial response, stable disease, and progressive disease groups were 30.1, 20.9, 18.5, and 12.9 months, respectively. The difference was statistically significant (*p* < 0.05). Univariate and multivariate Cox regression analyses demonstrated that the prognostic factors affecting OS were distant metastasis, maximum tumor diameter, TACE duration, and alpha-fetoprotein (AFP) level (*p* < 0.05). The overall incidence of grade 3 and above AEs was 18.0% (70/389), and the overall safety was controllable.

**Conclusion:**

TACE-A significantly improved OS, PFS, and ORR in advanced HCC patients. At the first follow-up patients with a poor tumor response had a poor prognosis. Distant metastasis, maximum tumor diameter, TACE frequency, and AFP levels are important prognostic factors that affect OS in patients. AEs of combination therapy are safe and manageable.

**Clinical trial number:**

Chinese Clinical Trials Database (ChiCTR1900024030).

## 1 Introduction

New cases of hepatocellular carcinoma (HCC) in China account for approximately half of the world’s total, with approximately 1 million new HCC patients diagnosed every year. Moreover, HCC is the fourth most common malignant tumor and the second leading cause of cancer-related death (1–3). More than 60% of HCC cases are diagnosed in the middle and advanced stages when the chance of radical treatment is lost, especially for older patients with vascular invasion or distant metastasis, the disease progresses rapidly, the prognosis is poor, and the 5-year survival rate is low (4–6). Transarterial chemoembolization (TACE) is a common treatment modality for advanced HCC. However, the effects of TACE on tumors are bidirectional. Chemotherapeutic drugs directly damage the deoxyribonucleic acid and induce apoptosis. Ischemia and hypoxia caused by embolisms can inhibit tumor progression (7, 8). However, the curative effect declined (9). Furthermore, TACE may induce local hypoxia in tumors, leading to a series of adaptive changes in the transcription and expression of hypoxia-responsive genes in tumor cells. Additionally, TACE induces vascular endothelial growth factor (VEGF) expression and promotes neovascularization (10, 11), leading to tumor recurrence and metastasis. Apatinib is a new molecularly targeted anti-angiogenic drug that can selectively inhibit VEGF-2 receptors, thereby inhibiting the growth of tumor blood vessels and producing anti-tumor effects (12). Apatinib has been used to treat gastric, breast, and other cancers (13, 14).

A phase II clinical trial confirmed the efficacy of apatinib against HCC (15). Compared with TACE alone or single-agent apatinib, several studies have demonstrated that TACE combined with apatinib (TACE-A) is feasible and effective for the treatment of patients with advanced HCC (16–18). However, most previous studies were retrospective, single-center, small-sample studies, and the level of evidence was limited. This study aimed to investigate the safety and efficacy of TACE-A in the real-world treatment of advanced HCC. The prospective, large-sample, and multi-center nature of this study will undoubtedly provide better evidence-based medical evidence, to overcome limitations of previous studies.

## 2 Materials and methods

### 2.1 Patient information

The data analysis in this study was based on the real world, conformed to the Declaration of Helsinki, and was approved by our Medical Ethics Committee (ethics number: 2019198). All patients were informed of the treatment details and signed a written informed consent form before treatment. Between January 2019 and June 2022, eight medical centers enrolled 423 patients with advanced HCC who received TACE-A treatment. A total of 389 patients were included in the study after 34 patients were excluded because of apatinib in less than a month (n=19) and incomplete follow-up data (n=15).

Moreover, HCC was diagnosed according to the American Association for the Study of Liver Diseases guidelines/European Association for the Study of Liver Diseases pathological examination or noninvasive criteria (19).

Inclusion criteria: (1) age ≥ 18 years old; (2) enhanced computed tomography (CT)/magnetic resonance imaging (MRI) examination displayed at least one measurable liver target lesion; (3) Barcelona Clinic Liver Cancer (BCLC) staging classification B/C; (4) liver function Child-Pugh score ≤ 7; (5) Eastern Cooperative Oncology Group (ECOG) score 0–1; (6) platelet count ≥ 50×10^9^/L, or can be corrected after treatment. The exclusion criteria were as follows: (1) diffuse HCC or tumor burden exceeding 70% of the whole liver; (2) complete occlusion of the main portal vein without compensation; (3) Child-Pugh score > 7; (4) previous TACE, radiotherapy, systemic therapy, or other anti-tumor therapies; and (5) severe comorbidities, including severe heart, lung, kidney, or coagulation dysfunction.

### 2.2 TACE procedure with apatinib

The TACE procedures and adverse event (AE) response strategies related to apatinib treatment at each medical center were consistent after unified training. Furthermore, TACE procedures were performed as described in our previous report on the technique (19). All TACE procedures were performed by two experienced interventional radiologists under local anesthesia using the traditional femoral approach. Conventional angiography was performed using a 5F RH catheter (Terumo, Tokyo, Japan), and a microcatheter (Terumo) was used for super-selective arterial cannulation of the tumor-feeding artery branch. Doxorubicin (China Hisun Pharmaceutical Co., Ltd.) and lipiodol (Garber, France) were thoroughly mixed and injected into the blood vessels of the tumor. Then 300–500um gelatin sponge particles (Ailikang Co., Ltd., Hangzhou, China) were given to supplement the embolism until the blood flow slowed down or nearly stagnated. The dosage of super-liquefied iodized oil was 5–20 milliliters, and the dosage of adriamycin was 30–50 mg. The actual dosage should be adjusted according to the patient’s liver function, the number and size of the tumors, whether the tumor is rich in blood supply, and the operator’s experience.

After obtaining informed consent from the patient and notifying them of the relevant precautions, the patient received oral apatinib (Jiangsu Hengrui Medicine Co., Ltd., Jiangsu, China) at 250 mg/day, 3 days after the first TACE. If a grade 3 AE occurred related to apatinib, the administration of apatinib was adjusted every other day. Dosing is interrupted if AE (grade ≥ 3) or those related to drugs such as gastrointestinal bleeding continue to occur after dose adjustment. The dose was restored to 250 mg/day when the AEs were alleviated or eliminated. Apatinib was permanently discontinued if AE of grade ≥ 3 or above occurred again.

### 2.3 Follow-up and study objectives

The last follow-up period was in June 2022. Each medical center adopted a consistent follow-up protocol. Patients were followed up every 4–6 weeks after TACE. Imaging examinations included plain chest CT, multiphase contrast-enhanced CT of the upper abdomen, and multiphase dynamic contrast-enhanced MRI. For patients with other discomforts, the doctor will provide corresponding imaging examinations according to the clinical symptoms. In case imaging examinations reveal tumor recurrence, new tumor, or residual tumor activity in the liver, and if the patient has no contraindications to TACE, repeat the procedure, that is, the principle of on-demand TACE. In case of the following conditions TACE was discontinued: liver function worsened to Child-Pugh class C (uncontrolled ascites, severe jaundice, overt hepatic encephalopathy, or hepatorenal syndrome), ECOG > 2, or if the target lesion continued to progress after two consecutive TACE treatments (20).

In terms of systemic treatment, patients continued to receive apatinib before on-demand TACE, and apatinib was discontinued on the day of TACE until 3 days after. All patients were administered apatinib regularly for 2 months after treatment initiation. When the patient was determined to have disease progression after two evaluations, or after TACE-A treatment, it no longer benefited. According to the patient’s wishes, TACE was combined with camrelizumab, sintilimab, and other immunotherapies, or changed to a second-line systemic treatment such as regorafenib.

The main objective of this study was to determine the median overall survival (mOS) of patients, defined as the time from the start of combination therapy to death from any cause, and median progression-free survival (mPFS), defined as the time from the start of TACE combination therapy to the first tumor progression or death from any cause. The prognostic factors affecting overall survival (OS) were also analyzed. The secondary objective was to determine the objective response rate (ORR) of patients at the first and second follow-ups. According to the modified response evaluation criteria for solid tumors (21), two senior radiologists (with more than 10 years of experience) evaluated the patients’ imaging data. The results were divided into complete response (CR), partial response (PR), stable disease (SD), and progressive disease (PD). If the two radiologists disagreed, they were evaluated by a radiologist with over 15 years of experience, and the evaluation result was considered the final result. Moreover, AEs were evaluated according to the National Cancer Institute’s “Common Terminology and Terminology Standards for Adverse Events, “ version 3.0.

### 2.4 Statistical analysis

Statistical analyses were performed using SPSS 22.0 statistical software (IBMCorp., Armonk, NY, USA) and R Language (version 4.1.2; R Foundation for Statistical Computing, Vienna, Austria; https://www.r-project.org/). The continuous variables conforming to the normal distribution are represented by mean ± standard deviation (x ± s), while those not conforming to the normal distribution are represented by the median and interquartile range [M (P25, P75)]. Survival curves were estimated using the Kaplan–Meier method, and mOS and mPFS were calculated. Univariate and multivariate Cox regression models were used to analyze prognostic factors associated with OS. Statistical significance was set at *p* < 0.05. significant.

## 3 Results

### 3.1 Baseline characteristics and treatment status

Among the 389 patients included in this study, 75 (19.3%) were BCLC-B stage patients and 331 (80.7%) C-stage patients. The proportion of patients with single and multiple lesions was 197 (50.6%) and 192 (49.4%), respectively. Tumor size was defined as 50 mm in 147 cases (< 50 mm) (37.8%) and 242 cases (≥ 50 mm) (62.2%) (**Table 1**). Among them, 124 patients (31.9%) had only portal vein tumor thrombus (PVTT), and three patients had both portal vein and hepatic vein tumor thrombi. 294 patients (75.6%) underwent two or fewer TACE procedures, while 95 patients (24.4%) required more than two on‐demand TACE treatments. Based on TACE-A treatment, 64 patients received ^125^I seed implantation in PVTT, of which 53 (82.8%) with branched tumor thrombus and 11 patients with main and branch tumor thrombus received ^125^I seed implantation of the branch tumor thrombus combined with camrelizumab. In addition, only 16 patients with a main PVTT were treated with camrelizumab. Eighty patients (63.0%) with vascular invasion underwent additional treatment. Of 109 patients with distant metastases, 21(19.3%) received additional immunotherapy (4 with sintilimab and 17 with camrelizumab). Moreover, among stage C patients, 35 patients received TACE-A combined with immunotherapy (toripalimab in 3 cases, sintilimab in 6 cases, and camrelizumab in 26 cases). A total of 136 patients received TACE-A treatment, including immunotherapy or ^125^I seed implantation, accounting for 35.0%.

**Table 1:** Patient baseline characteristics.

Characteristics	Level	N=389
Age (years)		55.9 ± 10.1
Gender	Male	339 (87.1%)
	Female	50 (12.9%)
BCLC	B	75 (19.3%)
	C	314 (80.7%)
Child-Pugh	A	358 (92.0%)
	B	31 (8.0%)
ECOG Score	0	120 (30.8%)
	1	269 (69.2%)
Metastasis	None	280 (72.0%)
	Have	109 (28.0%)
PVTT	None	262 (67.4%)
	Have	127 (32.6%)
AFP (ng/ml)	<400	213 (54.8%)
	≥400	176 (45.2%)
HBV	None	151 (38.8%)
	Have	238 (61.2%)
Tumor number	Single	197 (50.6%)
	Multiple	192 (49.4%)
Maximum tumor diameter (mm)	<50mm	147 (37.8%)
	≥50mm	242 (62.2%)
TBIL (g/L)		18.7 ± 9.4
ALB (μmol/L)		37.9 ± 5.7
ALT (U/L)		33 (23,49)
Cr (µmol/L)		60.4 ± 13.2
APTT (s)		12.5 ± 1.9
PLT (10^9/L)		142.9 ± 2.7
The number of TACE	≤2	294 (75.6%)
	>2	95 (24.4%)

ECOG, Eastern Cooperative Oncology Group; AFP, alpha-fetoprotein; TBIL, total bilirubin; HBV, hepatitis B virus; TBIL, total bilirubin; ALB, albumin; ALT, alanine aminotransferase; Cr, creatinine; APTT, activated partial thromboplastin time; TACE, transcatheter arterial chemoembolization.

In our cohort, of the 188 patients who experienced progressive disease (PD) on first‐line TACE‐A, 76 (40.4%) went on to receive subsequent immune‐checkpoint inhibitor therapy. Specifically, 44 patients received camrelizumab and 32 received sintilimab, either immediately upon PD (direct combination) or following a short interval on apatinib alone (sequential strategy).

### 3.2 Efficacy and survival analysis

**Figure 1:**
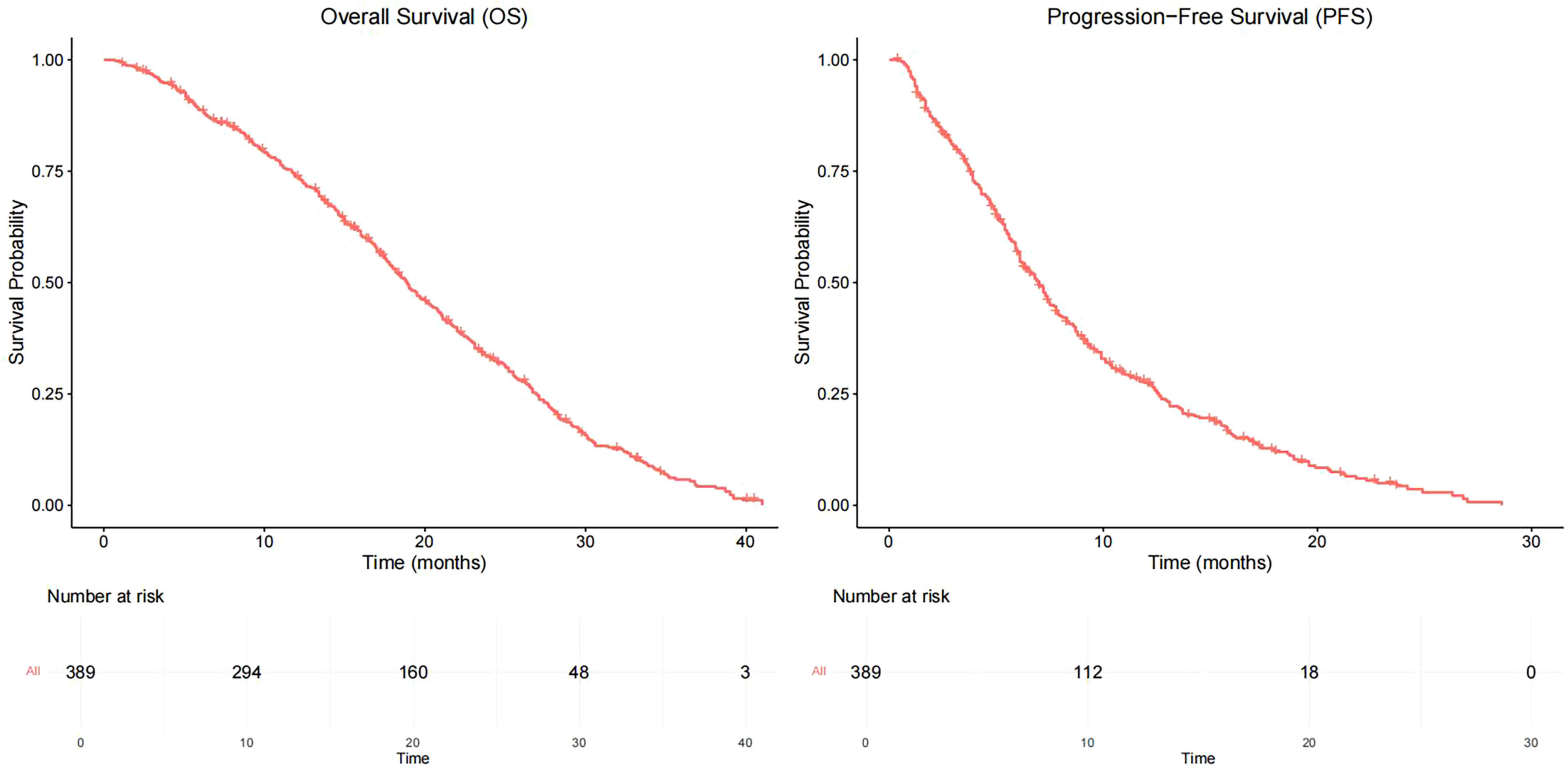
Kaplan-Meier curves for PFS and OS.

As of June 2022, 336 patients (86.4%) reached the study endpoint. The Kaplan–Meier curves of progression-free survival (PFS) and OS for all patients are displayed in **Figure 1**. The mPFS was 7.0 months (95% confidence interval [CI]: 6.3–7.7), and the mOS was 18.9 months (95% CI: 17.5–20.3).

The median time for the first follow-up/efficacy evaluation was 1.2 months, and no patients were lost to follow-up; of these, SD and PD patients were 24 (6.1%), 107 (27.5%), 206 (52.9%), and 52 (13.3%) patients were lost to follow-up, respectively. Moreover, the ORR was 33.6%. The patients were divided into 4 groups, the mOS of patients in CR, PR, SD, and PD groups were 30.1 months (95% CI: 20.4–39.8), 20.9 months (95% CI: 18.5–23.3), 18.5 months (95% CI: 16.5–20.5), and 12.9 months (95% CI: 10.4–15.4), the difference was statistically significant (*p* < 0.05). The median time of the second follow-up/response evaluation was 2.1 months, of which 51 cases (13.1%) were CR, PR, SD, and PD; 94 cases (24.2%), 108 cases (27.8%), and 136 cases (35.0%), respectively, and the ORR was 37.3% (**Table 2**). The two‐proportion z-test comparing ORR at the first (130/388 = 33.5%) versus second (145/389 = 37.3%) assessments yields a z-statistic of –1.099 with p = 0.272. This indicates no statistically significant difference in ORR between the two time points (p > 0.05).

**Table 2:** Objective response rate of patients at first/second efficacy.

Tumor response	The first efficacy	The second efficacy
CR	24	51
PR	106	94
SD	206	108
PD	52	136
ORR	33.6%	37.3%

CR, complete response; PR, partial response; SD, stable disease; PD, progressive disease; ORR, objective response rate.

### 3.3 Prognostic analysis

Univariate analysis demonstrated that six characteristics correlated with OS, including BCLC stage, presence of distant metastasis, maximum tumor diameter, TACE times, and alpha-fetoprotein (AFP) level. The aforementioned covariates were included in the multivariate Cox regression analysis using the direct entry method. The final results exhibited distant metastasis (95% CI: 1.52 (1.18–1.96), *p*= 0.001), the largest tumor diameter > 50 mm (95% CI: 1.51 (1.20–1.89), *p* < 0.001), TACE times < 2 (95% CI: 0.66 (0.51–0.85), *p*= 0.001) and AFP level ≥ 400ng/ml (95% CI: 1.50 (1.20–1.87), *p* < 0.001) were independent risk factors affecting OS (**Table 3**).

**Table 3:** Univariate and multivariate Cox analyses affecting OS of patients.

Characteristics	Univariate Cox regression		Multivariate Cox regression	
	HR(95%CI)	P value	HR(95%CI)	P value
Age	1.01 (1.00-1.02)	0.078		
Sex	0.95 (0.68-1.31)	0.744		
BCLC	1.60 (1.20-2.12)	**0.001**	1. 27 (0. 94-1. 72)	0. 116
CNLC				
Child-Pugh	0.89 (0.59-1.34)	0. 563		
ECOG	1.23 (0.97-1.56)	0.086		
Metastasis	1.53 (1.20-1.95)	**0.001**	1.52 (1. 18-1.96)	**0.001**
PVTT	1.21 (0.97-1.52)	0.096		
AFP	1.70 (1.36-2.12)	**<0.001**	1. 50 (1. 20-1.87)	**<0.001**
HBV	1.00 (0.80-1.24)	0.972		
Tumor number	0.93 (0.75-1.16)	0.52		
Maximum tumor diameter	1.64 (1.31-2.05)	**<0.001**	1.51 (1.20-1. 89)	**<0.001**
TBIL	1.01 (1.00-1.02)	0.171		
ALB	0.99 (0.97-1.01)	0.178		
ALT	1.00 (1.00-1.01)	0.183		
Cr	0.99 (0.99-1.00)	0.177		
APTT	1.02 (0.96-1.08)	0.495		
PLT	1.00(0.99-1.00)	0.195		
The number of TACE	0.66 (0.51-0.85)	**0.001**	0.66 (0.51-0.85)	**0.001**

All univariate variables (*p* < 0.05) were included in a multiple Cox regression analysis using the direct input method, and only the variables with *p* < 0.05 in the final model are presented; HR, hazard ratio; CI, confidence interval.

All values in bold in the table indicate statistically significant differences.

### 3.4 Survival analysis of patients with different characteristics

**Figure 2:**
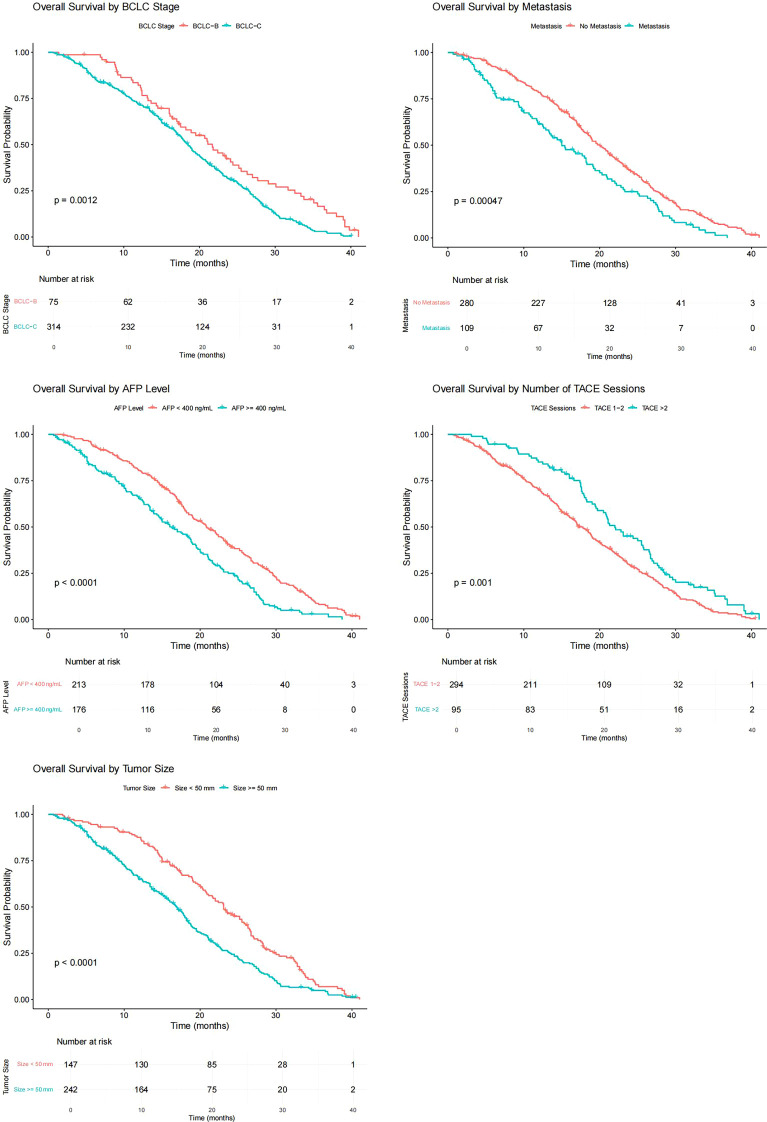
Kaplan–Meier curves for different variable groupings.

The variables with *p* < 0.05 in univariate Cox regression analysis included BCLC stage, presence or absence of distant metastasis, maximum tumor diameter, TACE times, and AFP level. The Kaplan–Meier curves of each variable grouping were plotted, and the mOS of each patient grouping was calculated. Among them, the mOS of BCLC-B and C patients were 21.7 months (95% CI: 17.73–25.67) and 18.5 months (95% CI: 17.16–19.84), respectively, and the B-stage patients were significantly better than C-stage patients (*p*= 0.001); the mOS of patients without distant metastasis and patients with distant metastasis were 20.0 months (95% CI: 18.41–21.59) and 15.0 (95% CI: 11.28–18.72), respectively, and the survival of patients with distant metastasis was worse (*p* < 0.001); the mOS of patients with APF levels < 400(ng/ml) and ≥ 400(ng/ml) were 20.8 months (95% CI: 18.53–23.07) and 16.3 months (95% CI: 13.58–19.02), respectively, and the difference was statistically significant (*p* < 0.001); the mOS of patients with maximum tumor diameter < 50 mm and ≥ 50 mm were 23.1 months (95% CI: 20.87–25.33) and 16.9 months (95% CI: 15.33–18.47), respectively, and patients with small maximum tumor diameter had a long survival period (*p* < 0.001); the mOS of patients who received more than two TACE treatments and those who only received two were 22.0 months (95% CI: 18.86–25.15) and 17.8 months (95% CI: 16.13–19.47), the difference was statistically significant (*p* = 0.001) (**Figure 2**).

### 3.5 Sensitivity analysis of apatinib compliance

In a sensitivity analysis stratifying patients by apatinib adherence, the irregular-medication group (n = 245) had a median OS of 17.6 months and a median PFS of 6.1 months, while the regular-medication group (n = 144) had a median OS of 17.2 months and a median PFS of 6.7 months. Kaplan–Meier curves with log-rank tests showed no significant difference in OS (p = 0.924) or PFS (p = 0.995) between the two groups.

### 3.6 Adverse events

During the treatment and follow-up periods, the most frequent treatment-related AE (TRAE) in patients receiving TACE-A was post-embolism syndrome, which was mainly characterized by fever, pain, nausea, vomiting, and fatigue, all of which improved after symptomatic treatment.

The overall incidence of TRAEs of grade 3 and higher was 18.0% (70/389 patients). Among them, 45 cases of blood-related grade 3 AEs, 8 cases of decreased white blood cell count, 29 cases of decreased platelet count, and 8 cases of decreased neutrophil count were present. Moreover, 25 cases of non-blood-related grade 3 AEs, including 10 cases of hypertension, 6 cases of abnormal liver function, 8 cases of proteinuria, and 1 case of diarrhea were observed. After all, patients received symptomatic treatment and dose reduction or withdrawal, all TRAEs resolved (**Table 4**).

**Table 4:** Adverse events from any cause.

AEs	TACE-A (N =389)	
	Any grade	≥ grade 3
Post-embolization syndrome		
Fever	182 (46.8%)	0
Pain	153 (39.33%)	0
Nausea and Vomiting	106 (27.25%)	0
Hand-foot skin reactions	166 (42.67%)	0
Fatigue	25 (6.43%)	2 (0.51%)
Non-blood-related adverse reactions		
Hypertension	91 (23.39%)	10 (2.57%)
Abnormal liver function	51 (13.11%)	6 (1.54%)
Diarrhoea	32 (8.23%)	1 (0.26%)
Proteinuria	30 (7.71%)	8 (2.06%)
Hoarseness	8 (2.06%)	0
Oral ulcer	9 (4.63%)	0
Rash	18 (4.63%)	0
Gastrointestinal hemorrhage	21 (5.40%)	0
Gastrointestinal reaction	45 (11.57%)	0
Blood-related adverse reactions		
Low white blood cell count	81 (20.82%)	8 (2.06%)
Platelet drop	149 (38.30%)	29 (7.46%)
Anaemia	114 (29.31%)	0
Increased alkaline phosphatase	32 (8.23%)	0
Decreased neutrophil count	86 (22.11%)	8 (2.06%)

## 4 Discussion

This study is a prospective, multi-center, large-sample real-world study, the mOS was 18.9 months (95% CI: 17.5–20.3), and the mPFS was 7.0 months (95% CI: 6.3–7.7). We further confirmed that the TACE-A treatment regimen effectively prolonged OS and PFS in patients with advanced HCC. In addition, we identified that patients with different tumor responses at the first follow-up had different prognoses. Patients who achieved a CR were considered to have a better survival benefit (30.1 months). However, the patients with PR and SD did not demonstrate a statistical difference (20.9 months vs 18.5 months). At the same time, the results of this study demonstrated that distant metastasis, tumor diameter > 50 mm, TACE time < 2 months, and high AFP levels were independent risk factors affecting OS, which was similar to previous research results (18, 22, 23).

Interestingly, PVTT was not the main factor affecting OS in this study, which contradicts previous reports (5). This may be due to the use of PVTT particle implantation combined with immunotherapy in the aforementioned patients. According to previous reports (24–27), 125I seed implantation and immunotherapy is an effective method for treating PVTT, which explains why patients with PVTT also achieved good curative effects. Yuan et al. reported that TACE-HAIC combined with targeted therapy and immunotherapy for hepatocellular carcinoma with portal vein tumor thrombus is sufficiently safe and leads to survival benefit. Previous studies have confirmed that the prognosis of tumor thrombi associated with the main portal vein is worse than that of branched tumor thrombi. In this study, 11 patients with branch and main tumor thrombi received simultaneous ^125^I seed implantation of the branch tumor thrombi and camrelizumab immunotherapy. Although seed implantation in branch tumor thrombi does not have a direct effect on the main tumor thrombus, it is internal radiotherapy that may enhance the effect of immunotherapy. Therefore, in real-world observations, we believe that even with PVTT, particle-combined immunotherapy can achieve a better curative effect, which may become a research direction in the future.

The results of the first randomized controlled trial of TACE-A were reported by Lu et al. (18). The long-term efficacy of the combination therapy is better than that of TACE alone and can prolong the PFS of patients. The ORRs at 3, 6, 9, and 12 months were 60%, 50%, 45%, and 35%, respectively, which were higher than those in our study. This may be because the number of patients with advanced disease enrolled in their study was much higher than that enrolled in our study (80.7% VS 11.9%, respectively). However, the dosage was also higher than that used in our study (500 mg/qd vs. 250 mg/qd), but there is no conclusion that high-dose apatinib can provide better survival benefits. In a real-world study of TACE-A for the treatment of advanced HCC (28), the mOS was 30 months, which is much higher than that reported in our study. Most patients (119/168, 70.8%) were treated with TACE-A in combination with other treatments such as arsenic trioxide, microwave ablation, and radioactive seed implantation. The proportion of patients with PVTT was low (45/119, 37.8%) and the proportion of patients with stage B disease was high (73/119, 61.3%). Additionally, the results of the aforementioned study demonstrated that combination therapy was an independent risk factor for survival. This seems to prove in another way that by providing additional treatment at the initial time and with early tumor staging, patients can achieve better survival. The mOS of the combined TACE group was 20 months, which was similar to our findings. However, the sample size in this study was only 103 (57.87%), and the advantages of the large sample size will undoubtedly provide better evidence-based medical evidence. In particular, some patients (63.0%) with vascular invasion were treated with ^125^I seed implantation or immunotherapy based on TACE-A, which is in line with the current concept of treatment for patients with vascular invasion. Therefore, based on TACE-A, further research on combined treatment strategies and potential benefits to patients should be conducted in the future.

The RESCUE trial demonstrated that apatinib combined with camrelizumab demonstrated good efficacy and safety as first- or second-line treatment for advanced HCC (15). Notably, most patients receive local therapy, including TACE, after disease progression. Therefore, conducting research on TACE-A or TACE combined with camrelizumab is important to further verify the application of this program in HCC.

The ORR of the first and second efficacy evaluations were not significantly different (33.6% VS 37.3%). However, the proportion of patients has increased, further confirming that patients with better tumor responses (CR and PR) at the first assessment can continue to benefit, whereas, the proportion of patients who achieved CR has increased. For patients with PD evaluated in the first assessment, the number increased during the second evaluation and they had a short OS. The above-mentioned finding indicates that among patients with poor tumor response in the first evaluation, changing the treatment strategy in advance may result in improved survival benefits. We also identified that in case patients did not benefit from previous treatment, most of them obtained improved survival benefits by the addition of other immunotherapies. This can be explained by the fact that TACE combined with targeted drug therapy not only inhibits the increase in VEGF but may also improve the immune microenvironment of some tumors according to previous studies (29, 30). The results of our study demonstrated for the first time that immunotherapy should be added in time to the treatments of patients with a poor tumor response, which may significantly improve the survival of such patients. However, the results require confirmation in a large-sample study.

This study identified that TACE-A has a good safety profile and tolerated AEs. Moreover, AEs related to embolism mainly include post-embolism syndrome (fever, nausea, vomiting, and pain). Furthermore, AEs associated with apatinib include hand-foot skin reactions, hypertension, fatigue, oral ulcers, proteinuria, and rashes. During subsequent immunotherapy, abnormal thyroid function, myocarditis, and pneumonia may be related to programmed cell death protein 1 inhibitors. Symptoms related to AEs were relieved or eliminated after symptomatic treatment or the temporary interruption of drug administration. Therefore, the findings of our study indicate that TACE-A is safe and controllable for the treatment of unresectable middle-advanced HCC patients (15, 18, 27, 28).

This study had some limitations. First, although this was a multicenter real-world study, 38.6% of the patients did not receive apatinib treatment regularly after the first TACE treatment because of personal problems, epidemic prevention, and control issues. This may have affected the clinical benefits and the results of this study. Second, although the treatment and follow-up plans were unified in each center, differences in the efficacy of TACE owing to the influence of the operator’s experience may still exist. Therefore, the conclusions of this study need to be confirmed through randomized controlled trials. Camrelizumab has not been completely unified even as the last second-line treatment. Furthermore, the sample size and stratification design in this study are also limited, which may result in insufficient statistical power to detect certain small but clinically meaningful differences.

## 5 Conclusion

In conclusion, TACE-A significantly improved patient survival and increased ORR. For patients with poor tumor response, second-line additional immunotherapy may be beneficial. Lastly, AEs were safe and controllable.

## Data Availability

The raw data supporting the conclusions of this article will be made available by the authors, without undue reservation. Technology Major Project of the datasets used and/or analyzed during the current study are available from the corresponding author upon reasonable request.
